# A Case Series of Angular Sign of Henle Fiber Layer Hyperreflectivity (ASHH): A Novel Optical Coherence Tomography (OCT) Biomarker

**DOI:** 10.7759/cureus.93823

**Published:** 2025-10-04

**Authors:** Konstantinos Flindris, Athanasios Kaliardas, Ioannis Koumpoulis, Ioannis Melissourgos

**Affiliations:** 1 Ophthalmology, General Hospital of Ioannina "G. Hatzikosta", Ioannina, GRC

**Keywords:** acute posterior multifocal placoid pigment epitheliopathy (apmppe), angular sign of henle fiber layer hyperreflectivity, angular sign of henle fiber layer hyperreflectivity (ashh), case series, choroidal rupture, oct biomarker, ocular trauma, optical coherence tomography (oct), photoreceptor cell dysfunction

## Abstract

The angular sign of Henle fiber layer (HFL) hyperreflectivity (ASHH) is a newly described optical coherence tomography (OCT) biomarker of acute macular photoreceptor injury. It appears on OCT as a hyperreflective band along the oblique HFL, extending from the outer plexiform layer (OPL) to the ellipsoid zone (EZ). ASHH occurs in macular disorders of ischemic, inflammatory, or mechanical/traumatic origin. This case series describes three patients with ASHH in distinct conditions and discusses its clinical relevance. A 24-year-old man with acute posterior multifocal placoid pigment epitheliopathy (APMPPE) presented with acute bilateral painless central vision loss and placoid macular lesions after a respiratory infection. OCT showed an ASHH indicative of photoreceptor disruption. A 28-year-old man sustained blunt ocular trauma from an ice projectile with commotio retinae. Early OCT revealed an ASHH in the macula, indicating acute photoreceptor axon injury. An 11-year-old boy sustained blunt ocular trauma from a thrown stone, causing a choroidal rupture. Acute OCT presented an ASHH, highlighting severe outer retinal damage. ASHH was identified in all three cases at presentation and co-localized with clinically affected macular regions. Serial OCT demonstrated reduction of hyperreflectivity over time in each case, with variable structural sequelae and favorable prognosis for the ultimate visual acuity. Across cases, documenting ASHH improved clinic-imaging correlation and provided a reproducible anchor for monitoring recovery. The HFL may be disrupted in various inflammatory, ischemic, and traumatic retinal conditions. In APMPPE, inner choroidal ischemia and inflammation lead to photoreceptor injury. In contusion injuries, force transmission causes photoreceptor axonal swelling and HFL disruption. ASHH typically resolves over weeks, often leaving outer retinal thinning or retinal pigmented epithelium changes with some visual sequelae. Recognizing ASHH as an OCT biomarker across diverse conditions may guide diagnostic procedures and therapeutic interventions.

## Introduction

Optical coherence tomography (OCT) has revolutionized retinal imaging by enabling high-resolution, cross-sectional visualization of retinal microstructure [1]. As OCT use has expanded, characteristic imaging signs have been identified that correlate with specific pathological processes [2]. The Henle fiber layer (HFL) consists of tightly packed, unmyelinated photoreceptor axons intertwined with the distal processes of Müller cells. These axons originate from cellular bodies of the photoreceptors in the outer nuclear layer and fan out radially toward the outer plexiform layer, whose inner one-third contains synaptic junctions, while the outer two-thirds correspond to the HFL itself [3]. One emerging finding is hyperreflectivity in the HFL of the macula, which comprises the obliquely oriented photoreceptor axons in the outer plexiform layer. Changes in HFL reflectivity can signal outer retinal disturbances and have been noted in various macular disorders. In particular, a newly described OCT feature termed the angular sign of Henle fiber layer hyperreflectivity (ASHH) has been proposed as a distinctive biomarker of acute photoreceptor disruption involving the HFL. This sign appears as a triangular or angular hyperreflective band in the macular OCT, typically extending from the outer plexiform layer through the photoreceptor zone, around the ellipsoid zone (EZ), in an acute setting of outer retinal injury [4-5].

Recent reports have documented ASHH across a spectrum of macular pathologies, suggesting a common structural endpoint of photoreceptor stress [6-8], including acute macular neuro-retinopathy (a microvascular ischemic injury to outer retina), dengue maculopathy (infection-related retinopathy), whiplash and contusion maculopathies (traumatic retinal injuries), acute placoid inflammatory diseases such as acute posterior multifocal placoid pigment epitheliopathy (APMPPE), and certain retinal vascular occlusive events. In each, ASHH is observed in the early phase, signifying acute outer retinal damage. However, recognition of this sign in clinical practice is still nascent. Its precise pathophysiology and full diagnostic implications are under active investigation.

In this report, we present a case series of three patients - encompassing an inflammatory chorioretinopathy and two distinct ocular traumas - all of whom demonstrated the ASHH on OCT during the acute phase of macular disruption. Through detailed multimodal imaging and clinical follow-up, we characterize the appearance and evolution of the ASHH in each case, correlate it with clinical findings, and assess visual outcomes. We further discuss the proposed pathophysiologic mechanisms underlying ASHH, its utility in diagnosis and monitoring of macular disease, and how this OCT sign can help distinguish outer retinal pathology across diverse etiologies. Our series underscores ASHH as an emerging OCT-based structural biomarker and highlights its relevance in retinal imaging and patient care.

## Case presentation

This study is a retrospective observational case series of three patients who developed acute macular pathology and were noted to exhibit the angular sign of HFL hyperreflectivity on OCT. The cases were selected from patients presenting to the Emergency Department of General Hospital of Ioannina “G. Hatzikosta” in Ioannina, Greece, during the past year. All patients underwent comprehensive ophthalmic examination, including best-corrected visual acuity (BCVA) measurement, dilated fundoscopy, and multimodal imaging. Imaging modalities included MultiColor fundus photography, fundus autofluorescence, and most critically, spectral-domain OCT of the macula. In one case, OCT angiography (OCTA) was also performed to evaluate chorioretinal circulation.

OCT imaging was performed using SPECTRALIS OCT (Heidelberg Engineering GmbH, Heidelberg, Germany), obtaining high-density macular scans through the fovea and areas of interest. Particular attention was given to identifying hyperreflective changes in the HFL and outer retina. ASHH was defined as a sharply demarcated, angular hyperreflective band in the HFL on OCT, spanning from the outer plexiform layer towards the photoreceptor EZ, in association with acute disruption of the outer retinal layers. Clinical data, including the context of injury or disease onset, treatments (if any), and follow-up findings, were collected from the medical records.

All patients gave informed consent for the use of their clinical data and images. Given the descriptive and retrospective nature of this case series, formal Institutional Review Board approval was not required or was exempted, and the study adhered to the tenets of the Declaration of Helsinki. Each case is detailed below in accordance with CARE case report guidelines, with identifying information omitted to maintain patient confidentiality.

Case 1: APMPPE in a 24-year-old male

A 24-year-old man with no significant medical history presented with a three-day history of painless subacute vision loss in both eyes. One month prior, he had experienced a flu-like febrile illness that resolved spontaneously. At presentation, BCVA was 20/65 in the right eye (OD) and 20/25 in the left eye (OS) in Snellen notation. Fundus examination of both eyes revealed multiple flat, cream-colored placoid lesions at the level of the retinal pigment epithelium (RPE) in the posterior pole, about 1 disc diameter in size with indistinct edges. These lesions were consistent with the clinical appearance of APMPPE. There was no significant vitritis or vasculitis. The optic discs were mildly hyperemic but without edema. Given the characteristic fundus and history of a recent systemic viral prodrome, an acute placoid inflammatory retinopathy was suspected.

**Figure 1:**
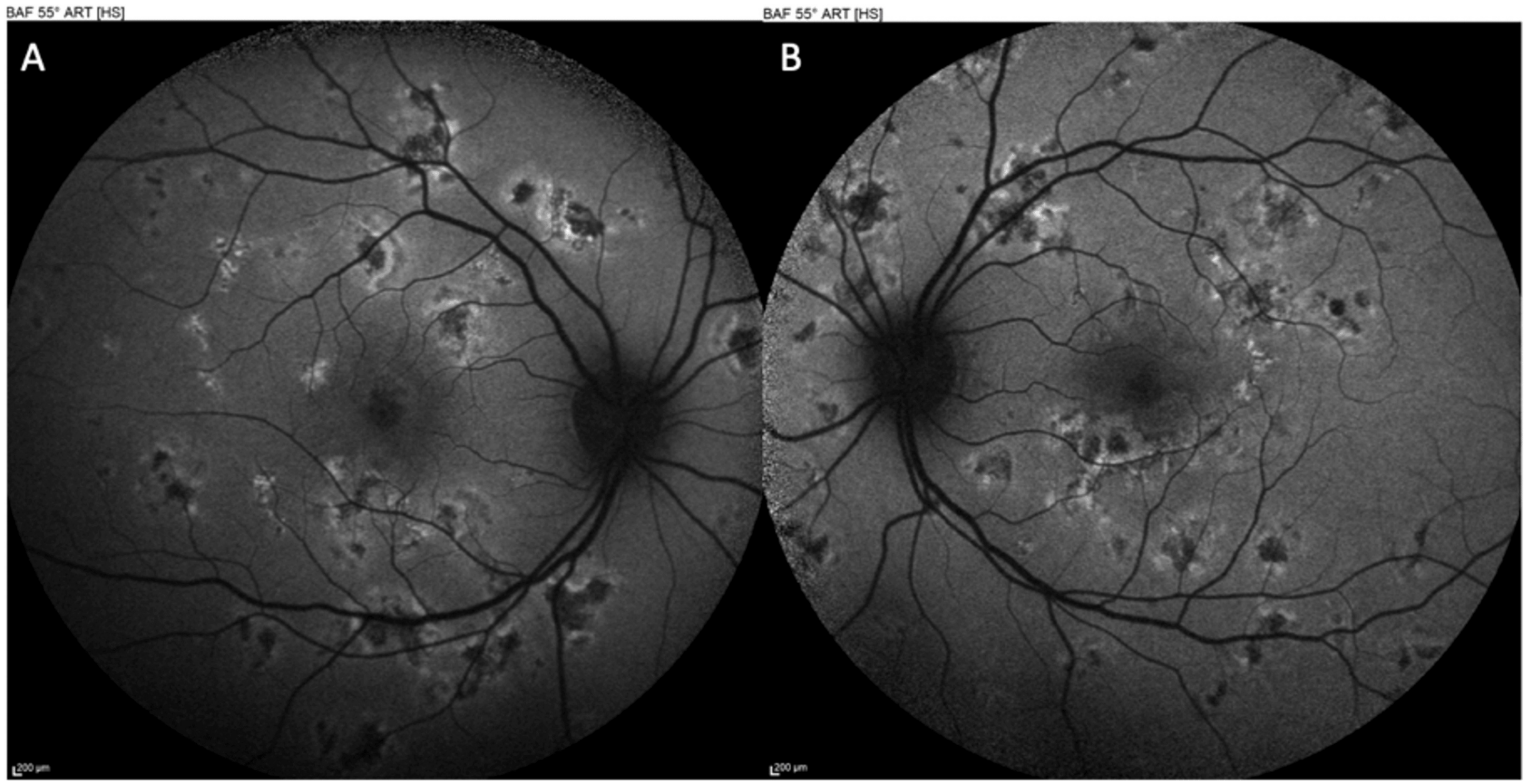
Fundus autofluorescence OD (A) and OS (B) at presentation, presenting hypoautofluorescent patches with subtle hyperautofluorescent rims, indicating acute retinal pigment epithelium (RPE) disturbance

**Figure 2:**
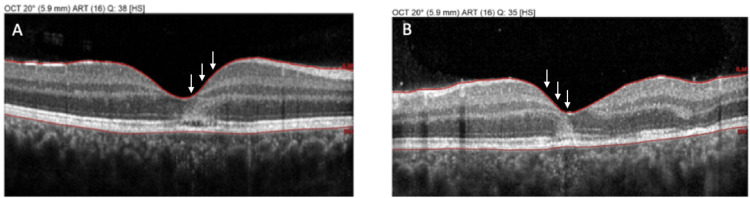
OCT OD (A) and OS (B) at presentation, demonstrating a converging hyperreflective band (ASHH) in the outer plexiform layer on either side of the fovea (arrows), indicative of acute photoreceptor disruption

**Figure 3:**
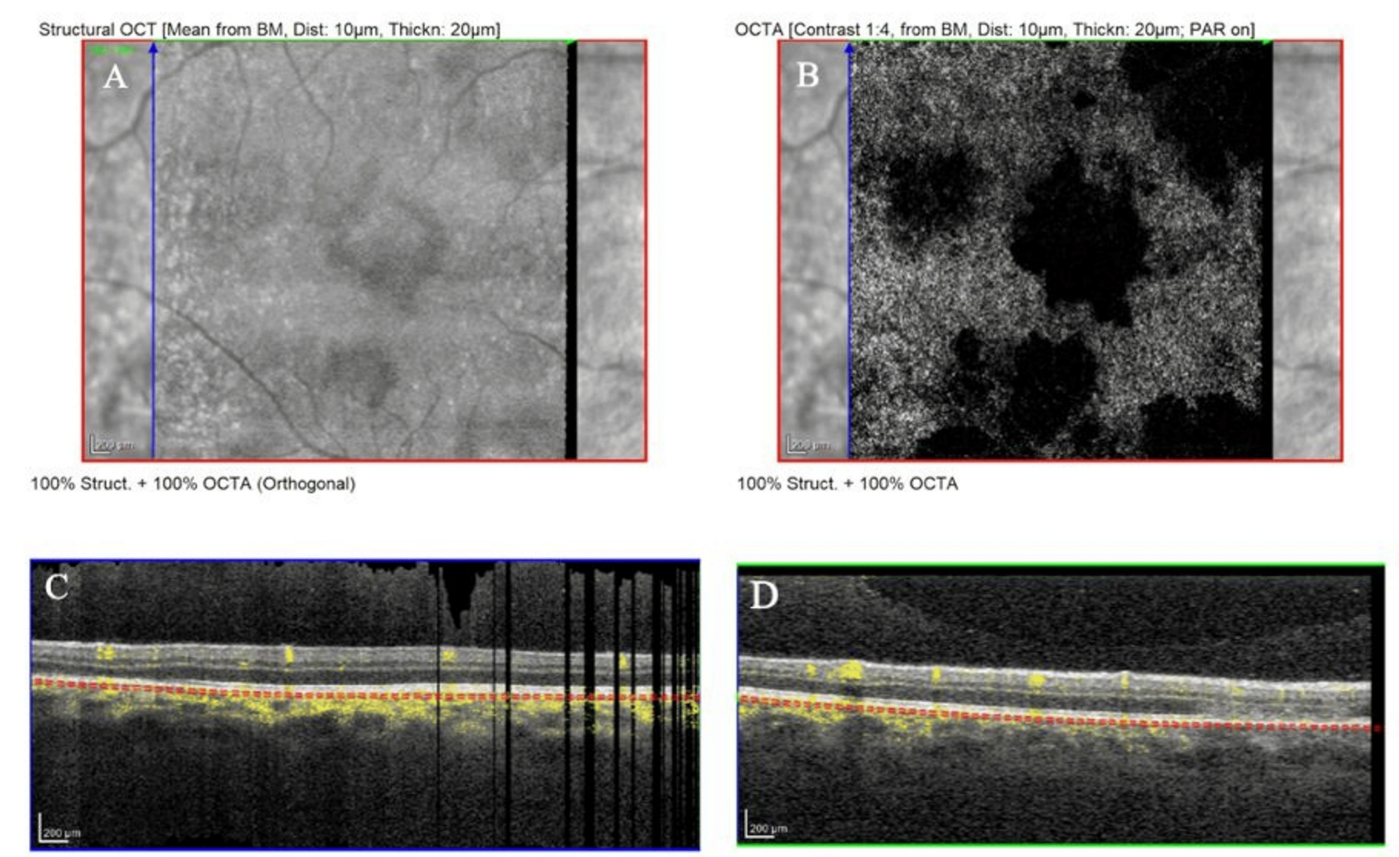
Optical coherence tomography angiography (OCTA) OD at presentation, revealing multiple patchy areas of flow voids at the level of the choriocapillaris corresponding to the placoid lesions (A, B), consistent with ischemia of the choriocapillaris underlying the photoreceptor damage (C, D)

Baseline multimodal imaging confirmed the diagnosis. Fundus autofluorescence showed the placoid lesions as hypoautofluorescent patches with subtle hyperautofluorescent rims, indicating acute RPE disturbance (Figure 1). Crucially, macular OCT scans through the fovea in both eyes demonstrated disruption of the outer retina corresponding to the placoid lesions. There was hyperreflective material at the level of the outer nuclear layer and RPE in lesion areas, with localized attenuation of the EZ band beneath each placoid lesion. At the foveal center of each eye, an angular configuration of hyperreflectivity was observed in the HFL - the ASHH sign (Figure 2). The HFL appeared as two converging hyperreflective bands in the outer plexiform layer on either side of the fovea, forming an unmistakable angular shape at the central fovea on the OCT cross-section. This ASHH finding indicated acute photoreceptor disruption at the fovea. No subretinal fluid or retinal detachment was present. Baseline OCTA was obtained, showing multiple focal areas of flow void in the choriocapillaris corresponding to the placoid lesions, consistent with choroidal ischemia underlying the photoreceptor damage (Figure 3). A thorough systemic work-up for infectious or other uveitic etiologies was unremarkable, supporting the diagnosis of idiopathic APMPPE. The patient was managed conservatively with observation (no acute immunosuppressive therapy), given that his central vision, although reduced, was still in a functional range and there were no signs of ocular or CNS complications.

Over the next two weeks, the patient experienced substantial visual improvement. By the two-week follow-up, BCVA improved to 20/30 OD and 20/20 OS, and the central scotomas resolved. On exam, the placoid lesions had faded, leaving faint RPE mottling in their place. Repeat OCT at two weeks showed marked structural recovery of the outer retina, as the previously noted outer retinal hyperreflectivity had largely cleared, the EZ band reappeared in areas that had been disrupted, and the foveal ASHH had resolved. Mild residual EZ irregularity and RPE undulations were present in a few spots, corresponding to healing lesions. Choroidal thickness, which was mildly increased during the acute phase, normalized on enhanced-depth OCT scans. By the three-month follow-up, the patient’s OD vision had further improved to 20/25, with only subtle pigmentary changes at the macula. No choroidal neovascularization or recurrence developed. This case highlights that in APMPPE, ASHH appears at the height of outer retina inflammation and photoreceptor injury, and it disappears as the inflammation resolves and photoreceptors recover, in parallel with visual restoration.

Case 2: blunt macular trauma from an ice projectile in a 28-year-old male

A 28-year-old man was referred after an ocular injury caused by a high-velocity ice projectile that struck his right eye during a work accident. He noted immediate pain and a central blur in the right eye following the trauma. On initial examination, BCVA in the right eye was 20/50, and 20/20 in the uninjured left eye. The anterior segment presented upper eyelid edema, mild conjunctival injection, and corneal abrasion, without hyphema. Extraocular movements were full, without diplopia or pain, and pupils were equal, round, and reactive to light bilaterally. Intraocular pressure (IOP) was 11 mmHg in both eyes. Dilated fundus examination of the right eye revealed signs of commotio retinae in the macula, a subtle gray-white discoloration of the fovea. There were no retinal tears or detachments, no significant subretinal hemorrhage was observed, and the optic nerve appeared normal. The diagnosis was contusion maculopathy due to blunt trauma.

**Figure 4:**
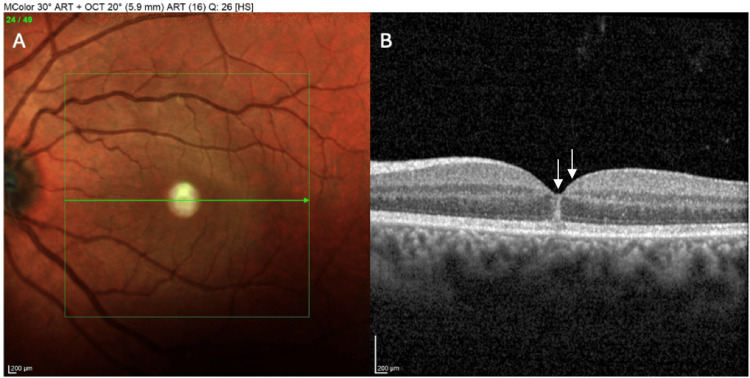
MultiColor fundus photograph OD (A), demonstrating a subtle gray-white discoloration of the foveal. Acute optical coherence tomography (OCT) (B) of the macula OD, showing a wedge-shaped hyperreflective lesion in the Henle fiber layer (HFL) at the fovea, consistent with the angular sign of HFL hyperreflectivity (ASHH) sign (arrows)

Acute OCT of the macula OD was performed to assess the extent of retinal injury and revealed a hyperreflective band across the outer retina at the fovea. Specifically, there was a wedge-shaped hyperreflective lesion in the HFL at the fovea, consistent with the ASHH sign (Figure 4). This angular hyperreflectivity spanned from the outer plexiform layer into the overlying photoreceptor layers at the central macula. Correspondingly, the underlying EZ and interdigitation zone were blurred or locally absent under the fovea, indicating acute photoreceptor disruption. Mild disorganization of the outer nuclear layer was present in the central macula, while the RPE appeared intact without obvious rupture or hemorrhagic detachment. These OCT findings confirmed that the blunt trauma had caused an acute injury to the photoreceptor layer and HFL, even though outwardly the retina only showed faint whitening. The left eye’s OCT was normal.

The patient was managed conservatively with antibiotic eye drops (tobramycin four times a day), antibiotic eye ointment (tobramycin two times a day), and artificial tears six times a day and was advised to rest and use protective eyewear. No surgical intervention was indicated since there was no macular hole or retinal tear. Over the ensuing month, the patient’s visual symptoms gradually improved. At a four-week follow-up, his right eye BCVA had recovered to 20/25. He reported resolution of the central blur and only minimal distortion. Follow-up OCT demonstrated resolution of the acute HFL hyperreflectivity, and the photoreceptor layer reconstituted, as the EZ band became continuous again through the fovea, with only a faint residual disruption at the center. The outer nuclear layer architecture was normalized, and no new findings were observed. Fundus examination at one month showed that the macula appeared clinically normal, without scars or pigmentary changes. This case illustrates that in contusion maculopathy, the ASHH can be an acute OCT marker of photoreceptor trauma. The disappearance of ASHH over weeks corresponded with anatomical and functional recovery, in line with the typically good prognosis of isolated foveal contusion injuries that do not cause structural breaks.

Case 3: blunt ocular trauma with peripapillary choroidal rupture in an 11-year-old male

**Figure 5:**
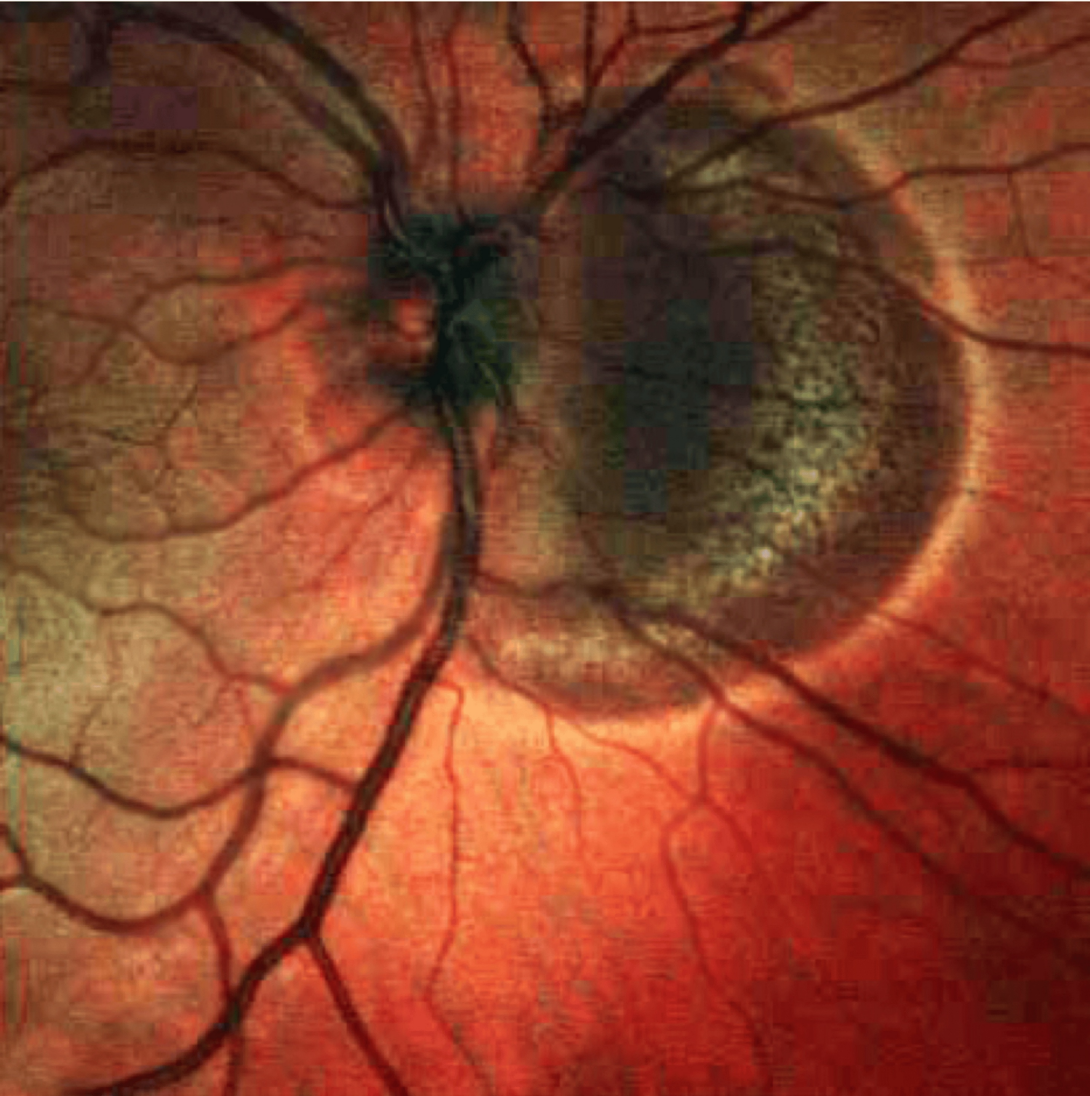
MultiColor fundus photography OD at presentation, exhibiting a crescent-shaped subretinal hemorrhage and an underlying choroidal rupture nasally of the optic disc

**Figure 6:**
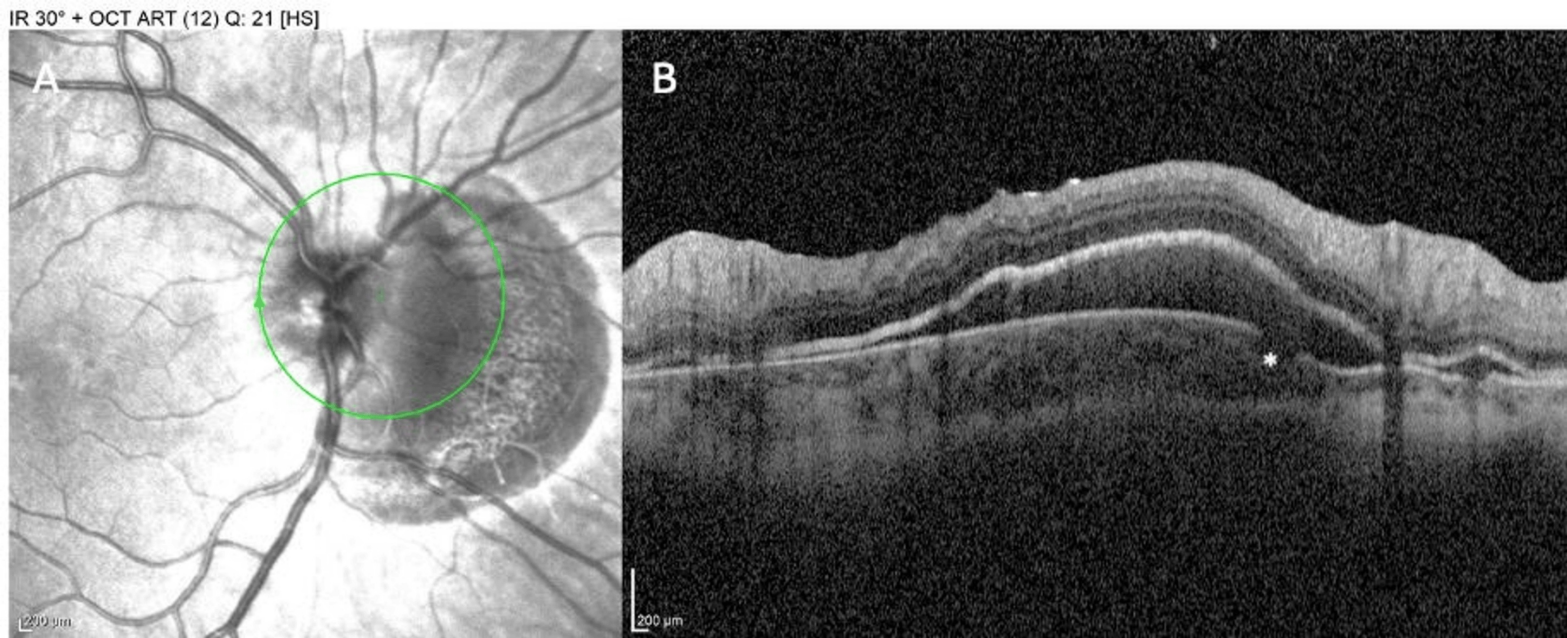
Infrared (IR) photography OD (A) and OCT retinal nerve fiber layer (RNFL) (B) at presentation, showing a peripapillary subretinal hemorrhage and an underlying choroidal rupture nasally of the optic, with traumatic rupture of the Bruch’s membrane (*)

An 11-year-old boy presented to the emergency department after being struck in the right eye by a thrown stone. He experienced immediate painful vision loss in that eye and nausea. On examination, his BCVA in the injured right eye was 20/50 with pinhole and in the left eye was 20/20. There was periorbital swelling and ecchymosis, conjunctival injection, corneal abrasion, and corneal edema without globe rupture. IOP was 35 mmHg OD and 12 mmHg OS. The dilated fundus examination of the right eye revealed a crescent-shaped subretinal hemorrhage and an underlying choroidal rupture nasally of the optic disc (Figure 5). The rupture appeared as a curvilinear whitish break in the RPE and choroid peripapillary (Figure 6). The optic disc was without overt damage.

**Figure 7:**
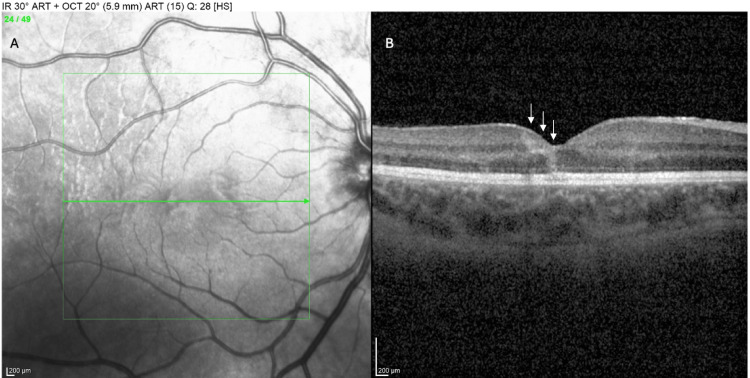
Infrared (IR) photography OD (A) and optical coherence tomography (OCT) of the fovea OD (B), presenting an oblique hyperreflective segment (angular sign of Henle fiber layer hyperreflectivity (ASHH)) in the outer plexiform layer/HFL (arrows), corresponding to acute photoreceptor axonal damage from the trauma

An urgent OCT of the right macula was obtained to evaluate the foveal architecture and extent of retinal damage. The OCT cross-sections through the fovea OD demonstrated an oblique hyperreflective segment in the outer plexiform layer/HFL (ASHH), corresponding to acute photoreceptor axonal damage from the trauma (Figure 7). The photoreceptor EZ layer was damaged across the fovea, and the outer nuclear layer was attenuated, signifying photoreceptor loss or dysfunction. Despite the severe injury, the inner retinal layers remained largely intact, and no full-thickness macular hole was present; thus, the structural damage was concentrated at the level of the outer retina.

Initial management involved antibiotic eye drops (tobramycin four times a day), antibiotic eye ointment (tobramycin two times a day), cycloplegic eye drops (cyclopentolate three times a day), antiglaucoma eye drops (timolol/dorzolamide two times a day), and artificial tears six times a day. Surgical intervention was not performed acutely; given the patient’s age and the location of the rupture, a decision was made to monitor for potential complications such as choroidal neovascularization (CNV) as the injury healed. Over four weeks of follow-up, the subretinal hemorrhage gradually absorbed, and the BCVA improved to 20/25 OD. Follow-up OCT showed that the previously ASHH in the fovea had diminished, with EZ restoration and without any RPE atrophy. The patient is monitored closely for the development of CNV. This case illustrates that in severe blunt ocular trauma with significant retinal damage, ASHH appears in the acute phase with a favorable prognosis for the functional and anatomical recovery of the fovea.

All three cases demonstrated the ASHH on OCT as an acute sign of macular photoreceptor injury, despite the disparate etiologies of their conditions. In each case, acute OCT imaging revealed a characteristic angular or wedge-shaped hyperreflective band in the macular outer plexiform layer corresponding to HFL, centered at or near the fovea. This sign was accompanied by disruption of the outer retinal layers (particularly the EZ) directly beneath the hyperreflective HFL region, confirming its association with photoreceptor damage. Notably, ASHH was documented in an inflammatory ischemic context (case 1, APMPPE) and in two traumatic mechanical injury contexts (Cases 2 and 3), underscoring that different pathogenic mechanisms can produce a similar OCT manifestation when the photoreceptor axons in Henle’s layer are acutely affected. Ancillary imaging supported the outer retinal nature of these injuries: for instance, in APMPPE (Case 1), there were corresponding choriocapillaris flow voids on OCTA and RPE autofluorescence changes, whereas in the trauma cases, there were clinical signs of commotio retinae or hemorrhage. However, regardless of cause, the OCT appearance of an ASHH was a unifying feature indicating acute outer retinal stress.

Evolution of the ASHH over time correlated with clinical improvement. In all cases, the visual recovery was favorable, and the ASHH was a transient phenomenon. At initial presentation, all cases showed a pronounced HFL hyperreflective angle at the fovea on OCT, and patients had moderate vision loss. With conservative management and over a follow-up period of weeks, the ASHH in all cases completely resolved on subsequent OCT scans. Concomitantly, the outer retinal layers reconstituted to a significant degree, evidenced by the reappearance or smoothing of the EZ line, and visual acuity improved to near-normal levels. These findings suggest that resolution of the ASHH is linked with recovery of photoreceptor structure and function. Herein, across the series, ASHH with normalization of HFL reflectivity indicated reversible photoreceptor injury.

Comparative analysis of the three cases also highlights certain distinguishing features in OCT and clinical findings. The APMPPE case showed multiple lesion loci with ASHH specifically at the fovea, accompanied by RPE changes and choroidal perfusion deficits, aligning with a primary choriocapillaris ischemic etiology. The traumatic cases showed ASHH in the context of mechanical retinal shock: one with purely functional disturbance (contusion) and one with anatomic disruption (rupture). In the pure contusion (Case 2), aside from ASHH and photoreceptor layer changes, the retina’s structural integrity was maintained (no breaks or gross lesions), and it healed completely. In the rupture case (Case 3), ASHH co-existed with a frank break in the RPE/choroid and hemorrhage, portending more serious injury. However, the OCT findings evolved in the same way as in Case 2. Hence, the common imaging denominator was the angular hyperreflectivity in Henle’s layer during the acute phase, reinforcing that ASHH is a reliable indicator of acute outer retinal injury.

## Discussion

This case series demonstrates that the ASHH is a consistent OCT marker of acute macular photoreceptor injury across markedly different conditions. The presence of ASHH in an inflammatory chorioretinopathy (APMPPE) and in blunt trauma-induced maculopathies supports the notion that this sign reflects a final common pathway of outer retinal disruption. Essentially, when the cone photoreceptor axons in the HFL undergo acute stress or damage - whether from ischemia, inflammation, or mechanical force - they produce a characteristic OCT signal in the form of angular hyperreflectivity. Our cases align with recently published observations of ASHH in various macular diseases, consolidating the idea that Henle’s layer is a vulnerable structure that reacts in a stereotyped way to acute photoreceptor insults [9].

The pathophysiology underlying ASHH is still being elucidated. Anatomically, Henle’s fiber layer is unique to the macula: photoreceptor axons run obliquely from the foveal center outward, surrounded by Müller cell processes [10]. This radial, angular arrangement likely explains the appearance of the sign. In a healthy eye, the reflectivity of HFL on OCT is influenced by the directional optical properties of the photoreceptors and can be relatively dim or isointense unless viewed at specific angles or disrupted by pathology [11]. When an acute injury occurs, several changes may contribute to HFL hyperreflectivity: edema or swelling of photoreceptor axons, gliosis or Müller cell activation, and misalignment of the normally well-ordered fiber layer, all of which can increase light scatter [12]. The result is a bright, wedge-shaped reflection on the OCT. The “angular” configuration often refers to the appearance of two symmetric hyperreflective slopes meeting at the fovea, especially evident in bilateral or symmetric lesions, as in our APMPPE case, or a solitary wedge pointing to the foveal center, as in some unilateral lesions. ASHH has been interpreted as a sign of HFL ischemia, for example, in acute macular neuroretinopathy and acute fovealitis, it is thought that a deep retinal capillary plexus insult leads to localized ischemia of the outer retina, manifesting as HFL hyperreflective wedges [13]. In inflammatory placoid diseases like APMPPE, the ischemic insult is at the level of the choriocapillaris beneath the photoreceptors, but it similarly results in acute photoreceptor dysfunction and HFL involvement [14]. In trauma, direct mechanical shock likely causes acute shear stress and metabolic compromise to photoreceptors and HFL [15]. Despite differing triggers, the OCT phenotype is analogous - indicating that ASHH is an optical signature of acute photoreceptor distress.

Recognizing ASHH has practical clinical implications for diagnosis and monitoring. Diagnostically, the presence of an ASHH on OCT should prompt clinicians to consider acute outer retinal processes (Table 1). For instance, in a patient with unexplained mild vision loss but an OCT showing a foveal ASHH, one should inquire about recent systemic symptoms (to consider an acute placoid outer retinopathy), or a history of trauma or systemic inflammation (to consider contusion injury or acute macular neuroretinopathy), even if the fundus exam is subtle [16]. In our Case 2, the commotio retinae was subtle clinically, but the OCT clearly showed ASHH and photoreceptor disruption, confirming a contusion diagnosis and delineating the extent of injury. Thus, the ASHH can be more sensitive than fundoscopy in detecting photoreceptor injury [17]. Furthermore, ASHH can help distinguish outer retinal involvement from inner retinal ischemic injuries: for instance, paracentral acute middle maculopathy (PAMM) causes hyperreflective bands in the inner nuclear layer (due to deep capillary plexus infarcts) but would not produce an ASHH, whereas acute macular neuroretinopathy (which affects outer retina) does show ASHH [18]. In our APMPPE case, identification of ASHH supported the acute nature of photoreceptor involvement, helping differentiate it from chronic or infectious lesions that might not show this acute OCT change. As a result, ASHH serves as a specific marker for acute photoreceptor layer pathology, guiding correct etiologic attribution and appropriate management [19].

**Table 1:** Conditions related with the ASHH sign ASHH: angular sign of Henle fiber layer hyperreflectivity Sources: [16-19]

Inflammatory conditions	Acute macular neuroretinopathy (AMN), acute posterior multifocal placoid pigment epitheliopathy (APMPPE), acute retinal pigment epitheliitis, acute fovealitis, acute annular outer retinopathy (AAOR), paraneoplastic autoimmune retinopathy
Ischemic conditions	Central retinal vein occlusion, central retinal artery occlusion, sickle cell retinopathy, idiopathic retinal vasculitis, Purtscher’s retinopathy, chronic cocaine use
Traumatic conditions	Whiplash maculopathy, contusion maculopathy, handheld laser-induced retinopathy

From a monitoring and prognostication standpoint, the course of ASHH on serial OCTs provides insight into retinal recovery [20]. In this series, all cases showed resolution of ASHH on follow-up OCT, corresponding to nearly complete restoration of the outer retinal architecture and excellent visual outcomes. This suggests that ASHH is a reversible phenomenon likely representing functional impairment (edema or transient injury) rather than permanent loss [21]. In general, a disappearing ASHH with reappearing EZ line is a positive sign, whereas disappearing ASHH with persistent EZ loss and thinning indicates photoreceptor death [22]. Consequently, tracking ASHH could have prognostic value: for example, in acute placoid diseases, the extent of HFL hyperreflectivity might correlate with the area of outer retinal ischemia and predict which regions will recover [23]. Indeed, emerging studies have started to evaluate the prognostic significance of acute HFL hyperreflectivity, finding correlations with visual outcomes and recovery times [24]. Clinicians can use this information to set expectations and tailor follow-up. In our traumatic cases, early identification of ASHH and its extent informed us about the magnitude of photoreceptor injury, and subsequent OCTs helped monitor the fovea and visual recovery.

It is also important to differentiate ASHH from other causes of HFL hyperreflectivity on OCT to avoid confusion. Chronic conditions can lead to hyperreflective material within Henle’s layer, such as RPE plume, exudates (macular star in optic disc edema or hypertensive retinopathy) or hemorrhages (underlying etiologies include: retinal vein occlusion, Valsalva retinopathy, Terson’s syndrome, intracranial hemorrhage, venous disorders, lacquers cracks associated with pathological myopia, macular neovascularization, polypoidal choroidal vasculopathy) [25]. Moreover, vertical hyperreflective lesions in vitreoretinal lymphoma, Cytomegalovirus (CMV) retinitis, vitreomacular reactions, and macular holes should be recognised as different causes of HFL hyperreflectivity on OCT [26]. The ASHH, by contrast, is an acute, transient intrinsic hyperreflectivity of the tissue, and is uniquely associated with concurrent photoreceptor layer disruption. In practice, correlating the OCT with fundus findings will usually clarify the cause of any HFL hyperreflectivity [27-28]. In our series, the absence of intraretinal exudation or chronic changes, and the clear temporal association with an acute event, supported that the observed HFL hyperreflectivity was indeed the ASHH of acute photoreceptor injury.

Our report contributes to the growing literature by providing detailed longitudinal documentation of ASHH in both inflammatory and traumatic scenarios. Nonetheless, there are limitations. The sample size is small (reflecting the rarity of some of these events), and this is a descriptive study without quantitative analysis of the hyperreflectivity or retinal layer thickness. We did not perform en face OCT analysis, which might better delineate the topographic shape of ASHH (wedge or donut-shaped patterns around the fovea). We also did not have OCT angiography data for the trauma cases; it would be interesting in future cases to see if there is deep capillary plexus flow impairment corresponding to ASHH in trauma, analogous to the flow deficits seen in APMPPE. Despite these limitations, the consistency of the findings across cases bolsters the validity of ASHH as a real phenomenon and not an artifact.

In summary, ASHH appears to unify a pathoanatomic response of the macula to acute injury. The HFL’s radial layout and relationship with both retinal and choroidal circulation make it a sensitive indicator of outer retinal health. When viewed in conjunction with other multimodal imaging and clinical data, ASHH enriches our understanding of disease processes: for example, it visually links conditions like APMPPE and contusion maculopathy by revealing that both involve an acute insult to the same retinal compartment. Ongoing research is needed to further clarify the cellular events that produce ASHH and to quantify its utility. Future directions may include using automated OCT analysis to detect ASHH and measure its area or intensity, correlating those metrics with visual outcomes, and exploring if any interventions can modulate the course of ASHH. Moreover, as high-resolution and directional OCT techniques evolve, we may gain even more insight into HFL changes.

## Conclusions

The ASHH is an emerging OCT biomarker that signifies acute structural injury to the macular photoreceptors and their axons in Henle’s layer. Through this case series of an acute placoid inflammatory retinal disease and two forms of blunt trauma, we have illustrated that ASHH can manifest across diverse macular injuries, serving as a common indicator of outer retinal damage. In each case, ASHH correlated with the onset of acute pathology, and its subsequent resolution mirrored the healing process and final visual outcome. These findings highlight ASHH’s potential utility in both diagnosing acute macular conditions and monitoring their course. Recognizing ASHH on OCT can alert clinicians to the presence of an acute outer retinal insult even when fundus signs are subtle, allowing timely and appropriate management. Moreover, as a unifying sign, ASHH underscores that different etiologies - inflammatory, ischemic, or traumatic - share a convergent impact on the photoreceptor layer that can be detected noninvasively. In the broader context of retinal diseases, ASHH is stepping forward as a meaningful structural biomarker that enhances our understanding of macular injury and recovery. As OCT technology and research into retinal injury continue to advance, ASHH is poised to become an integral part of the retinal imaging lexicon, aiding in the distinction of outer retinal pathologies and in the refinement of prognostic expectations across a range of macular disorders.
